# A new type low-cost, flexible and wearable tertiary nanocomposite sensor for room temperature hydrogen gas sensing

**DOI:** 10.1038/s41598-020-58965-w

**Published:** 2020-02-07

**Authors:** Deepak Punetha, Manoranjan Kar, Saurabh Kumar Pandey

**Affiliations:** 1 Sensors and Optoelectronics Research Group (SORG), Department of Electrical Engineering, Indian Institute of Technology Patna, Bihar, 801103 India; 2 Department of Physics, Indian Institute of Technology Patna, Bihar, 801103 India

**Keywords:** Environmental impact, Sensors

## Abstract

This paper reports on reduced graphene oxide (rGO), tin oxide (SnO_2_) and polyvinylidene fluoride (PVDF) tertiary nanocomposite thick film based flexible gas sensor. The nanocomposite of 0.90(PVDF) − 0.10[x(SnO_2_) − (1 − x)rGO] with different weight percentages (x = 0, 0.15, 0.30, 0.45, 0.6, 0.75, 0.90 and 1) have been prepared by the hot press method. Chromium (Cr) has been deposited on the surface by using E-beam evaporation system, which is used as electrode of the device. Crystal structure, morphology, and electrical characteristics of the device have been explored for the technological application. A correlation between crystallinity, morphology, and electrical properties with these thick films has also been established. The device has been tested at different hydrogen (H_2_) gas concentration as well as at different response times. A superior response of 0.90(PVDF) − 0.10[0.75(SnO_2_) − 0.25 rGO] nanocomposite thick film has been observed. Hence, this composition is considered as optimized tertiary nanocomposite for the hydrogen gas sensor application. The sensor response of 49.2 and 71.4% with response time 34 sec and 52 sec for 100 PPM and 1000 PPM H_2_ gas concentration respectively have been obtained. First time a new kind of low cost and flexible polymer based nanocomposite thick film gas sensor has been explored.

## Introduction

Hydrogen (H_2_) is the promising clean energy carrier for the future power generation due to its inexhaustible, abundant and portable nature. It possesses unique characteristics such as; low ignition energy (0.01 mJ), wide explosive concentration range (4–75 vol%), large flame propagation velocity, and high heat of combustion (142 kJ/g). Some additional features includes low molecular weight, high energy content and its combustibility without emitting any harmful gases, which make it an ideal choice for an alternative energy source^1,2^. One of the effective way to produce hydrogen is by means of zero carbon strategies such as nuclear power, solar, wind, and fossil fuels. Moreover, hydrogen finds its application in semiconductor processing, metal smelting, petroleum extraction, glassmaking, and chemical industry due to its strong reducing properties. Further, hydrogen can be employed for biomedical, environmental protection and seismic surveillance for indicating certain type of bacterial infection, detecting environmental pollution, etc. However, its uses are restricted due to its volatile and extremely flammable nature. Because a small leak of hydrogen from any system to the atmosphere can become very dangerous and sometimes cause a conceivable explosion. Hence, the detection of hydrogen in any system is a frontline research problem and challenge for the researchers. However, colorless, odorless, small in size, and tasteless nature of H_2_ makes it difficult to detect by human organs. Therefore an artificial H_2_ sensor with superior performance is essential for safety concern^3^.

In this regard, many metal oxide semiconductors such as ZnO, TiO_2_, WO_3_, SnO_2_, etc. have been used as the sensing layer^4^. Among these semiconducting metal oxides, tin oxide (SnO_2_) has been widely used as H_2_ gas sensor^5^. It behaves as n-type semiconductor and requires high power consumption and high operating temperatures (200–500 °C) for sensing applications^4^. The above limitation of high temperature has been overcome by carbon materials and, they are very effective at low-temperature gas sensing.

In other hand, graphene being a highly attractive and intensively investigated material has drawn considerable research interest for gas sensing applications due to its unique thermal and electrical properties^6^. The reduced graphene oxide is prevailing over the different derivatives of graphene due to its remarkable characteristics such as excellent response characteristics; chemical stability, large specific surface area, high carrier mobility, and good mechanical strength. All of these features of rGO makes it an ideal candidate for gas detection^7^.

Moreover, in the modern era, the focus of the researcher is oriented towards flexible gas sensor having good sensing ability at room temperature (RT). This flexible sensor (i.e. electronic skin) can be attached to the human body for detecting and sensing the pollutant gases^8^. There are various flexible materials that can be utilized as membrane formation. Polyvinylidene fluoride (PVDF) is a piezoelectric material having good UV and thermal stability, good mechanical strength, excellent chemical resistance and membrane formation features. It also has potential applications towards energy conversion such as energy harvesters and micro electrical-mechanical devices^9,10^. Hence, the above literature survey motivates to develop a flexible and high sensitive H_2_ gas sensor. By considering all the aspects, a tertiary nanocomposite based flexible hydrogen gas sensor has been fabricated by using rGO, SnO_2_, and PVDF to operate at room temperature. It is interesting to note that there is/are no report/s on rGO/SnO_2_/PVDF nanocomposites as a H_2_ gas sensor. Hence, the 1^st^ time such a sensor is reported for the H_2_ gas sensing applications. The interdigitated electrode of Cr metal has been deposited by using E-beam evaporation system. Various characterization tools have been used to determine the different properties of the thick film. The sensor has been tested inside a controlled gas chamber with I~V source meter for different gases and for different gas concentration at room temperature. It is observed that the proposed sensor is easy to prepare (low cost) and flexible (polymer) in nature and, exhibits excellent H_2_ gas sensing at room temperature. Hence, the present study opens a new window for accomplishing a polymer based tertiary nanocomposite H_2_ gas sensor.

## Experiment Details

**Figure 1:**
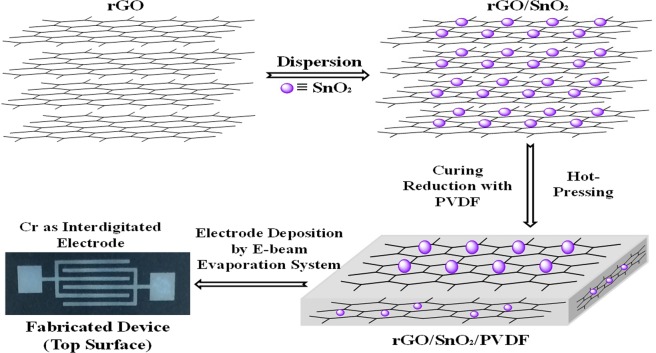
Schematic of the fabrication process steps of rGO/SnO_2_/PVDF nanocomposite gas sensor.

The nanocomposite thick films of 0.90(PVDF) − 0.10[x(SnO_2_) − (1 − x)rGO] with different weight percentages (x = 0, 0.15, 0.30, 0.45, 0.6, 0.75, 0.90 and 1) have been prepared by the hot press method. The processing steps have been shown in Fig. 1.

**Figure 2:**
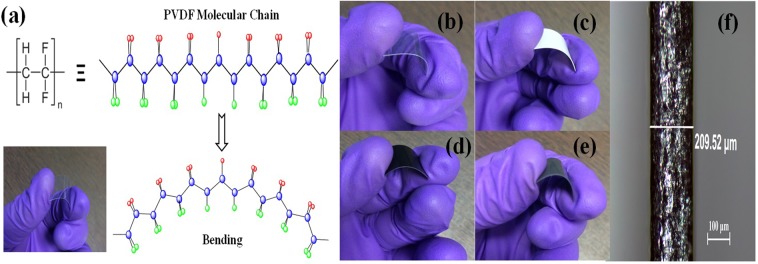
(**a**) Schematic illustration of PVDF molecular chain, Photographs of **(b)** PVDF, **(c)** SnO_2_, **(d)** rGO/PVDF, **(e)** rGO/SnO_2_/PVDF at bending position, and **(f)** transverse optical microscope image of rGO/SnO_2_/PVDF thick films (thickness ~209.52 μm).

The mixture of rGO:SnO_2_:PVDF has been prepared by mortar pestle. The mixture was kept in a stainless steel die and pressed at 180 °C under a load of 5 tonnes for 10 minutes by using a hot press machine. The temperature of the die was brought down to room temperature by running cold water and then the stress was released. Interdigitated pattern (electrode) of Cr was deposited by using E-beam evaporation system at 0–5 Å/sec deposition rate and chamber pressure of ~ 4 × 10^–6^ mbar. The rGO/SnO_2_/PVDF thick film sensor with Cr electrode was flexible in nature. The photograph of a typical sensor is shown in Fig. 2. The photograph was taken by folding the sensor to demonstrate its flexible nature. The thickness of composite film has been observed by an optical microscope which is found to be ~209.52 μm as indicated in Fig. 2(f).

The crystallinity of the PVDFnanocompositefilmwas characterized by the Rigaku TTRAX III X-ray Diffractometer (Cu-Kα (1.542 Å) within the 2θ range of 05–70°. The X-ray tube voltage (V) and current (I) were 50 kV and 100 mA respectively. Raman spectra were recorded in ambient condition by employing Raman spectroscopy (AIRIX Corp., Model: STR-750) method with He-Ne laser at wavelength 632.8 nm. The Fourier transform infrared (FTIR) spectra (PerkinElmer spectrum 400) of thick films were recorded in attenuated total reflectance (ATR) mode. The surface morphology of the sensing layer has been carried out by employing the Field Emission Scanning Electron Microscopy (FESEM) technique with the help of Zeiss GeminiSEM 500. The FESEM micrographs have been obtained at a magnification of 50 kX.

**Figure 3:**
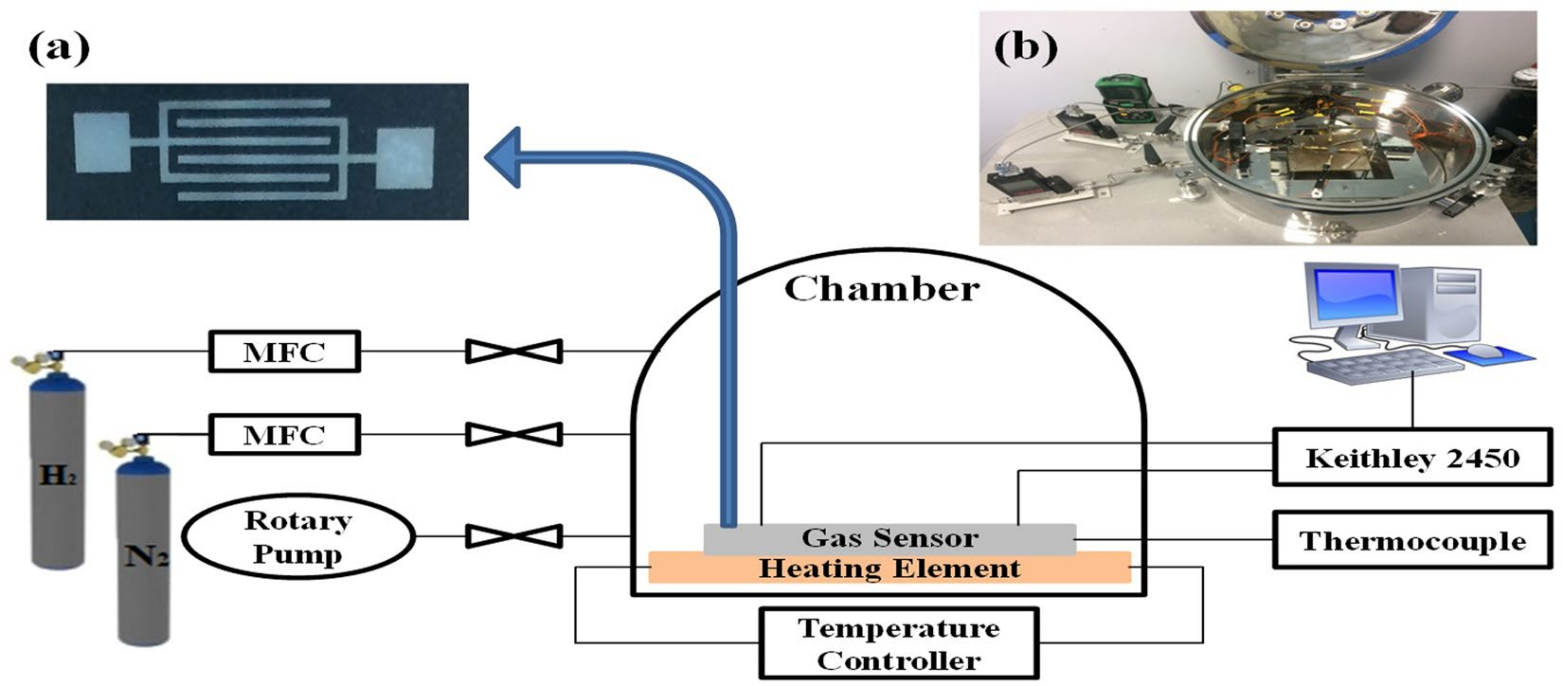
Schematic diagram of the gas chamber for sensing of gas in a controlled environment with (inset) **(a)** optical image of the fabricated sensor and **(b)** Real image of the gas chamber measurement system.

Here, the gas sensing principle is based on the change of resistance of the material because of the electronic and chemical interaction in between sensing layer and gas molecule. The chemical interaction comprises the target gas adsorption on the surface of catalyst then migration to the surface of PVDF based nanocomposites, which outcomes in the exploration for the target gas. The sensing and electrical properties of the sensor have been tested by using the Keithley 2450 IV source meter. The testing and analysis have been performed inside the gas chamber in a controlled atmospheric condition. To flow the gas inside the chamber, two Mass Flow Controllers (MFCs) having flow capacity of 10 SCCM and 1000 SCCM were used. Figure 3 shows the gas chamber system while inset on it shows the fabricated device and the experimental setup. The gas response of the sensor is defined by^4^:

1 $${\rm{Sensor}}\,{\rm{Response}}\,( \% )=\frac{{R}_{a}-{R}_{g}}{{R}_{a}}\times 100$$

where *R*_*a*_ and *R*_*g*_ represents the resistance of the gas sensor in the absence and presence of the gas respectively.

## Results and Discussion

**Figure 4:**
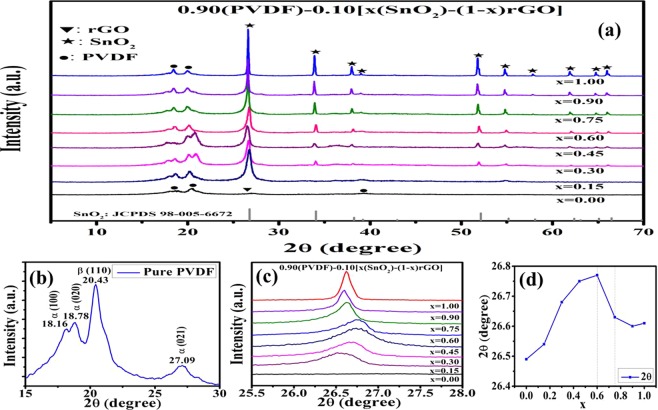
XRD pattern of PVDF based sensor, **(a)** 0.90(PVDF) − 0.10[x(SnO_2_) − (1 − x)rGO] with different weight percentages (x = 0, 0.15, 0.30, 0.45, 0.6, 0.75, 0.90 and 1), **(b)** enlarge view of XRD pattern from 15–30 degree to show the α and β phases of PVDF, **(c)** enlarge view of XRD pattern in the range of 25.5° to 28°, and **(d)** Composition versus highest intensity peak position in the XRD pattern (correspond to SnO_2_ phases).

Figure 4(a) depicts the XRD patterns of PVDF, rGO/PVDF, SnO_2_/PVDF and rGO/SnO_2_/PVDF nanocomposite films. The peaks correspond to the PVDF, rGO and SnO_2_ phases are marked as ‘●’, ‘▼’ and ‘★’ respectively. The observed peaks in the XRD pattern at 26.78°, 34.05°, 38.24°, 42.96°, 52.13°, 55.19°, 58.09°, 62.38°, 65.08°, and 66.46° are indexed to respectively (110), (011), (020), (120), (121), (220), (002), (130), (112), and (031) crystal planes of SnO_2_. It is well matched with JCPDS 98-005-6672 for SnO_2_. The peaks at 18.16°, 18.78°, and 27.09° are due to the non-polar α-phase while the peak at 20.43° related to the polar β-phase of PVDF which are marked in Fig. 4(b). It is observed that the intensity of the XRD peaks corresponds to α- phase of PVDF increases and peaks correspond to β-phase decreases with the increase in SnO_2_ concentration in the composite. The similar kind of behavior of SnO_2_/rGO composite has been previously reported in the literature^11,12^. Above changes of the PVDF XRD patterns in rGo/SnO_2_/PVDF nanocomposites reveal the incorporation of rGO and SnO_2_ into the PVDF matrix and change the structure of PVDF due to surface interaction. The wide peak at around 26.58° corresponds to the plane (002) for rGO is visible for x = 0 (absence of SnO_2_). But it is difficult to distinguish in the XRD pattern of rGO/SnO_2_/PVDF, because of its coincidence with SnO_2_ peaks. The similar result has been reported by other group^13^. For better understanding, the effect of SnO_2_ and rGO incorporation into the PVDF matrix, XRD pattern for the range of 25.5° to 28^°^ diffraction angle has been magnified and shown in the Fig. 4(c). The shifting of XRD peak with the weight percentage of rGO and SnO_2_ has been observed. This was due to the stretching of bonds, which could be due to the interaction of H-atom of PVDF and the oxygen of functional groups of rGO and SnO_2_^12^. The shifting of the peak as a function of composition has been shown in Fig. 4(d). The XRD peak shifts to higher angle upto the composition x = 0.6 and, it shifts to lower angle with the further increase of x (>0.6). Hence, it is assumed that the optimum composition will be at x = 0.6 ± 0.15. Hence, it is interesting to note that the maximum gas sensing action has been observed for x = 0.75 composite, which is discussed later. It is assumed the agglomeration of rGO and SnO_2_ for the composition x > 0.75.

**Figure 5:**
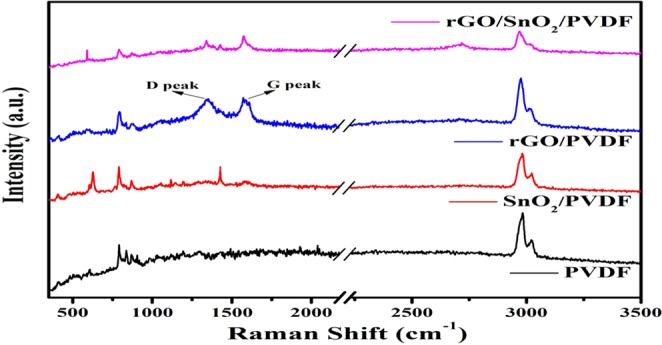
Raman spectra of PVDF and its nanocomposite with rGO and SnO_2_.

Raman spectra can clearly distinguish the carbon framework in the composite, thus Raman spectroscopy has been carried out in the spectral range of 300–3500 cm^−1^. Raman spectra of PVDF, SnO_2_/PVDF, rGO/PVDF, and rGO/SnO_2_/PVDF nanocomposites are shown in Fig. 5 which displays the three important characteristic peaks of rGO, named as the G band (the tangential mode of graphitic structure), D band (disorder-induced band) and the G_0_ (or 2D) band. It also compares Raman spectra of rGO/PVDF and rGO/SnO_2_/PVDF in 1st order region. Both composites demonstrated peaks of carbonaceous materials at 1350 cm^−1^ (D band) and 1592 cm^−1^ (G band), which is nearly identical to the other articles^12,14^. The Raman spectra are analyzed to support the results obtained from the analysis of the XRD results.

As a proportion, there is no Raman signal in the range of 1000–2500 cm^−1^ for PVDF. The D band/G band intensity ratio of rGO/PVDF and rGO/SnO_2_/PVDF was found to be 0.97 and 0.94 respectively. It confirms the further reduction of graphene oxide through the hot press method. The deformation generated by the amorphization of graphite has been perceived to cause enhancement in the relative intensities of the D to G bands, as a result of a lowering in the sp^2^ domain^15^. Due to breaking of symmetry, impregnated SnO_2_ NPs made the graphene layers extra wavy in rGO/SnO_2_/PVDF attributed to stronger π− π interaction, ultimately leading to reduction of sp^2^ domain size of graphene layers^16^. In the Raman spectrum of rGO/PVDF, the D, G and 2D peak positions ensure the rGO formation. This outcome shows that the chemical groups and rGO with defects have relocated to integrated graphene with high aspect. Raman spectrum of undoped SnO_2_ NPs demonstrates various characteristic bands of SnO_2_ in the low-frequency region, i.e., 424, 480 (E_g_), and 629 (A_1g_). All these peaks are almost suppressed in the rGO/SnO_2_/PVDF nanocomposite due to the presence of highly intense peaks in the rGO and PVDF sheets. Raman bands obtained at 791 cm^−1^ and 837 cm^−1^ in PVDF films coincide with α and β-phases and required to identify the phases of PVDF^17,18^. Distinctly, the PVDF film is governed by the characteristic α-phase band at 791 cm^−1^ and a weak band at 837 cm^−1^ compatible to the β-phase. For further understanding of α and β-phases evolution in PVDF, the FTIR spectroscopy has been employed. Also, the XRD pattern and Raman analysis are supported by the FTIR study.

**Figure 6:**
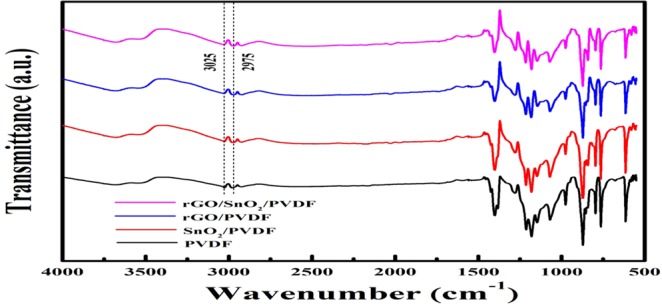
ATR-FTIR spectra of PVDF and its nanocomposition with rGO and SnO_2_ for the region of 500–4000 cm^−1^.

**Figure 7:**
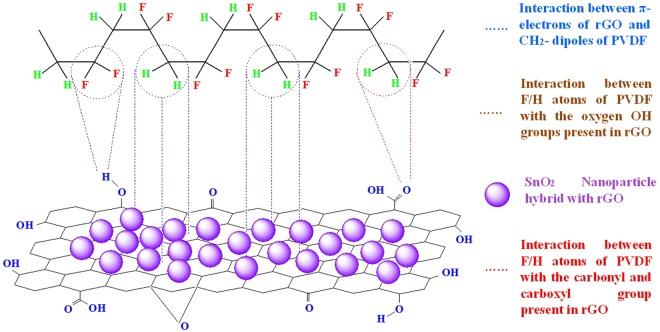
The proposed interaction mechanism between rGO, SnO_2,_ and PVDF.

The changes in molecular structure of PVDF based thick film have been observed through FTIR spectra as shown in Fig. 6. This tool is used in attenuated total reflectance mode to calculate qualitative analysis of functional groups present in the surface of PVDF, rGO/PVDF, SnO_2_/PVDF, and rGO/SnO_2_/PVDF nanocomposite thick films. The most significant aspect of the spectrum of primitive PVDF is the existence of sharp peaks nearby 874, 1180 and 1398 cm^−1^, which attributes to the stretching vibration of C–F, deformation vibrations and asymmetric stretching of C–H bond, respectively^12,19^. The band at 839 cm^−1^ corresponds to the mixed mode of CF_2_ asymmetric stretching and CH_2_ rocking. The absorption peaks observed at 2975 and 3025 cm^−1^ corresponds to symmetric and asymmetric vibration of CH_2_ respectively^20^. These observed absorption peaks have been retained in all other samples. Due to oxygen functional group present in rGO/PVDF, SnO_2_/PVDF, and rGO/SnO_2_/PVDF nanocomposite thick film, some other absorption bands also have been observed at 572 cm^−1^ (Sn-O-Sn stretching), 1067 cm^−1^ (C-O-C stretching), 1215 cm^−1^ (C-O stretching), and 1596 cm^−1^ (O-H bending)^21,22^. A wide peak has been observed at 3505 cm^−1^, which corresponds to the O-H stretching of carboxyl functional group. Two peaks have been observed at 760 and 974 cm^−1^ which is corresponding to α-phase of PVDF. This α-phase of PVDF is suppressed thus enhances the β-phase with the increase of rGO concentration in the nanocomposite. The β-phase enhance in PVDF due to graphene oxide has been reported by other groups^12^. This characteristic correlates with the results obtained in the XRD analysis. In proportion to the primitive PVDF membrane, a broad peak at around 3450–3500 cm^−1^ is noticed in rGO/PVDF, SnO_2_/PVDF, and rGO/SnO_2_/PVDF specimen. The residual oxygen containing functional groups present in the rGO interact with PVDF nanomaterials and hybridize with SnO_2_. Between the PVDF and rGO molecules, three types of interaction occur^23^. First, the interaction between π- electrons of rGO and CH_2_ dipoles of PVDF, second is the interaction between F/H atoms of PVDF with the OH group present in rGO, and third is the interaction between F/H atoms of PVDF with the carbonyl and carboxyl group present in rGO. The proposed interaction mechanism between rGO, SnO_2,_ and PVDF has been shown in Fig. 7.

**Figure 8:**
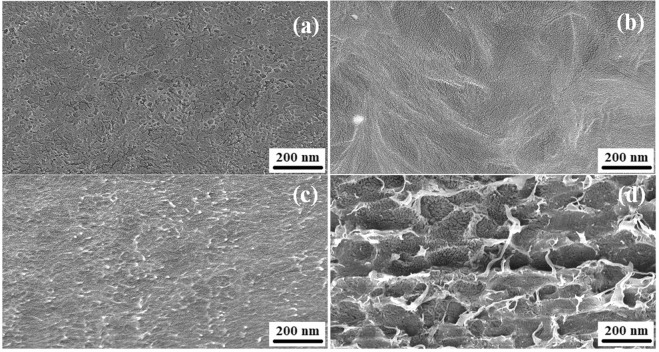
FESEM micrographs of **(a)** PVDF, **(b)** rGO/PVDF, **(c)** SnO_2_/PVDF, and **(d)** rGO/SnO_2_/PVDF.

The surface morphology of PVDF, rGO/PVDF, SnO_2_/PVDF and rGO/SnO_2_/PVDF nanocomposite films have been characterized by the FESEM as shown in Fig. 8. Micrographs are found to be uniform. The rGO shows the wrinkled structure and it is observed that rGO and SnO_2_ nanomaterial is well dispersed in PVDF polymer.

## Gas Sensing Analysis

The adsorption mechanism in the above mentioned sensor is based on physisorption and chemisorption process. In the physisorption process, hydrogen atoms adsorbed on the surface of PVDF nanomaterial by van der Waals forces^24–26^. These van der Waals forces are weak in nature, which leads to a small variation in conductance for PVDF nanomaterial sensing layer. Whereas in the chemisorption process, due to the formation of covalent bond, it has strong van der Waals forces^27,28^. This chemisorption mechanism has been seen for rGO and SnO_2_ nano-composite with PVDF nanomaterial. When these nanocomposite based sensor is exposed to air, oxygen species adsorbed on material surface by occupying the electrons from conduction band to make anions of chemisorbed oxygen (O_2_^−^(ads)). It further results in the formation of space charge region, i.e. depletion region^29^.

2 $${{\rm{O}}}_{2}({\rm{air}})\to {{\rm{O}}}_{2}({\rm{ads}})$$

3 $${{\rm{O}}}_{2}({\rm{ads}})+{{\rm{e}}}^{-}\to {{{\rm{O}}}_{2}}^{-}({\rm{ads}})$$

When this rGO/SnO_2_/PVDF nanocomposite exposed to reducing gas such as hydrogen, the adsorbed hydrogen molecules or atom interacts with the oxygen anions present in the surface; given by following reaction^30^:

4 $${{\rm{2H}}}_{2}+{{{\rm{O}}}_{2}}^{-}({\rm{ads}})\to 2{{\rm{H}}}_{2}{\rm{O}}+{{\rm{e}}}^{-}$$

Due to this charge transfer process, the electrons concentration increases leading to decrement in resistivity of the sensing layer.

**Figure 9:**
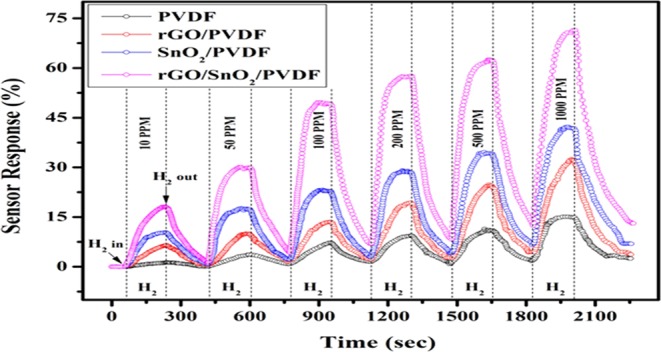
Sensor response of PVDF, rGO/PVDF, SnO_2_/PVDF, and rGO/SnO_2_/PVDF gas sensor for different H_2_ gas concentrations.

Figure 9 shows the sensor response (%) of PVDF, rGO/PVDF, SnO_2_/PVDF and rGO/SnO_2_/PVDF nanocomposites for different gas concentrations at room temperature. The gas response of the sensor has been tested for different concentrations (i.e. 10 PPM, 50 PPM, 100 PPM, 200 PPM, 500 PPM, and 1000 PPM) of H_2_ gas for above mentioned films. It has been observed that the gas concentration has a great impact on the sensor response where the response and recovery time is calculated as the time taken to reach 90% of its equilibrium value^31^. The chemisorption process takes more time than the physisorption process because of high energy requirement for the formation of covalent bonds^28^. This effect also has been observed for the present sample specimens.

**Figure 10:**
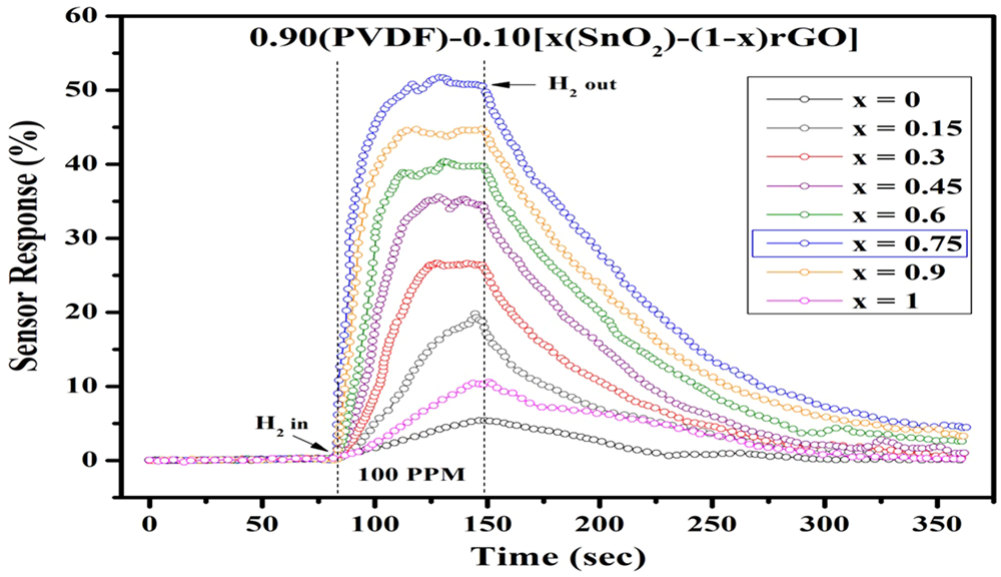
Sensor Response analysis for 0.90(PVDF) − 0.10[x(SnO_2_) − (1 − x)rGO] nanocomposite with different weight percentages (x = 0, 0.15, 0.30, 0.45, 0.6, 0.75, 0.90 and 1).

The sensor response for 0.90(PVDF)−0.10[x(SnO_2_)-(1-x)rGO] with different weight percentages (x = 0, 0.15, 0.30, 0.45, 0.6, 0.75, 0.90 and 1) nanocomposite thick film have been tested for 100 PPM gas concentration of hydrogen gas shown in Fig. 10. It has been observed that the above sample with weight percentage (x = 0.75) shows the highest sensor response among other PVDF based nanocomposite specimens. It justifies the results of XRD studies. As discussed from the XRD result, the gas sensing supposed to be maximum for x = 0.6 ± 0.15. The better sensing response observed in rGO/SnO_2_/PVDF nano-composite which leads to more active reaction sites is due to its high surface area characteristics. The sensor shows the superior results for 0.90(PVDF) − 0.10[0.75(SnO_2_) − 0.25 rGO] nanocomposite. The result shows the strong influence of rGO weight percentage on sensing performance. On increasing the rGO content upto x = 0.75, the sensor performance degrades, while further increase in rGO content leads to an increment in the graphene sheet, which encloses the SnO_2_ nanomaterial by wrapping the active sites^32^. Hence, 0.90(PVDF) − 0.10[0.75(SnO_2_) − 0.25 rGO] nanocomposition, has been considered as optimized compositions.

**Figure 11:**
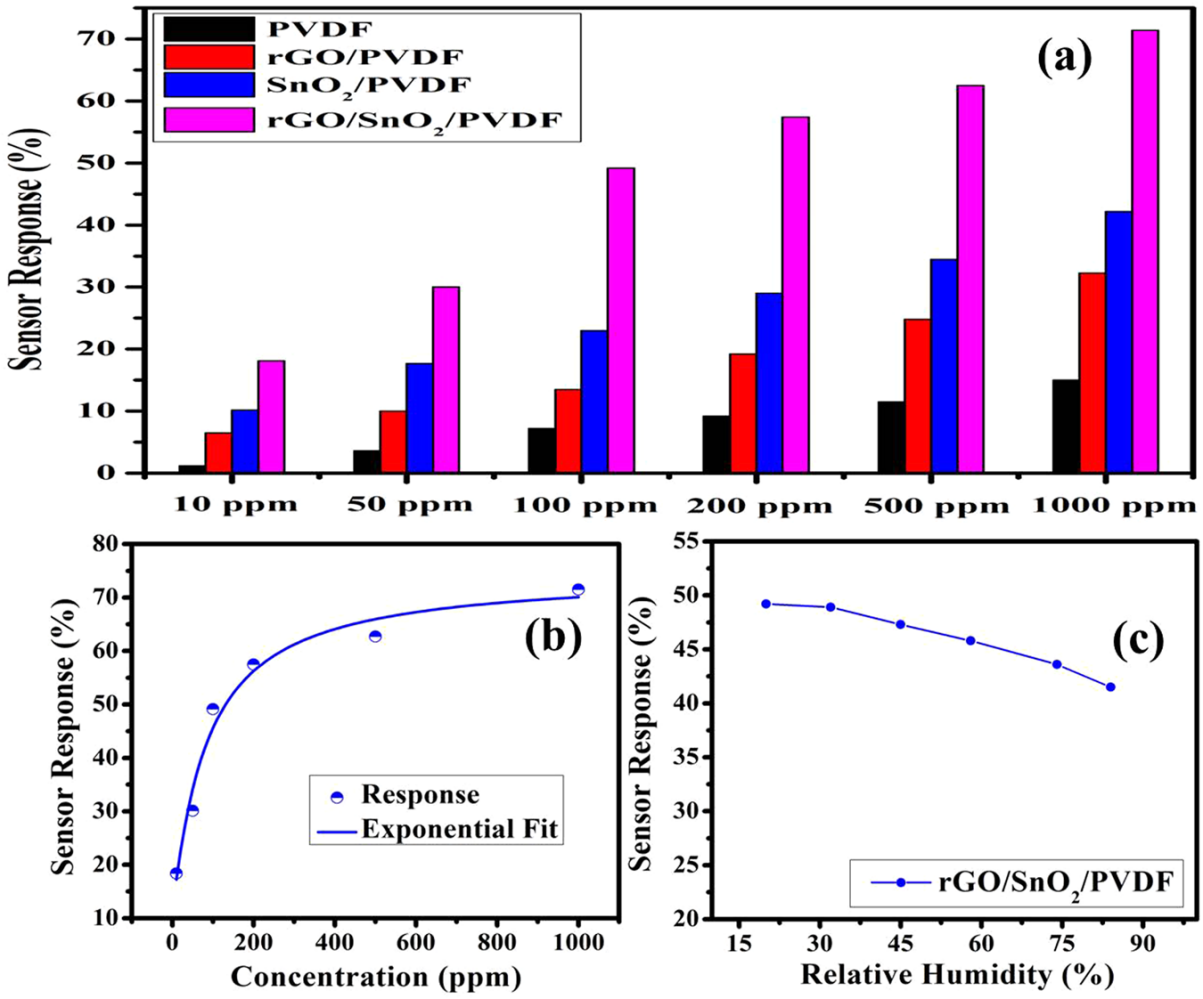
Gas sensing response, **(a)** For PVDF, rGO/PVDF, SnO_2_/PVDF, and rGO/SnO_2_/PVDF nanocomposite thick film towards different hydrogen gas concentration, **(b)** Response curve and the fitting analysis of the response of the 0.90(PVDF) − 0.10[0.75(SnO_2_) − 0.25 rGO] nanocomposite sensor for different H_2_ gas concentration, and **(c)** Sensing performance of the proposed nanocomposite sensor towards 100 PPM H_2_ exposure under different humidity conditions.

The comparative sensor response analysis for PVDF based nanocomposite material has been performed and shown in Fig. 11(a). The sensor response of SnO_2_/PVDF is better than rGO/PVDF, which signifies that the hydrogen has a high sticking coefficient for PVDF nanocomposite with SnO_2_ nanomaterial than rGO nanomaterial. From the response analysis, it is observed that the sensor response of the rGO/SnO_2_/PVDF nanocomposites sample is higher than other nanomaterials. It may be due to the fact that by making the nanocomposition of rGO/SnO_2_/PVDF, surface area of the sensing layer drastically increases thus escalate the gas response, which also has been observed from the FESEM micrographs. To check for the linearity of responses with H_2_ gas concentrations, the 0.90(PVDF) − 0.10[0.75(SnO_2_) −0.25 rGO] nanocomposite gas sensor was exposed to a wide range of H_2_ concentrations from 10 to 1000 ppm (Fig. 11(b)). It has been observed that the response shows the exponential behavior with respect to increase in H_2_ gas concentration. The similar behavior is also has been recorded in literature^33^. Figure 11(c) shows the response of proposed nanocomposite sensor towards 100 ppm H_2_ exposure with change in 20%, 32%, 45%, 58%, 74% and 84% relative humidity (RH). The sensor response decreases with the increase in RH %.

**Figure 12:**
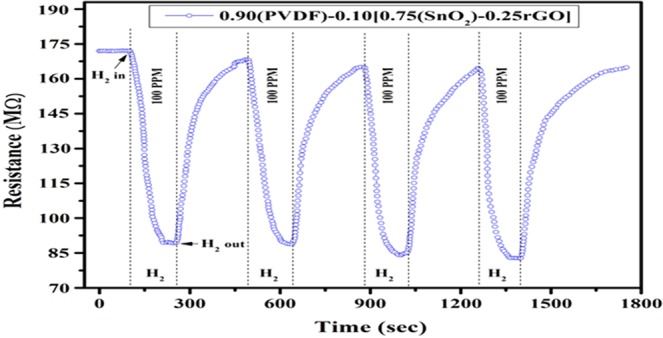
Baseline resistance of 0.90(PVDF) − 0.10[0.75(SnO_2_) − 0.25 rGO] nanocomposite gas sensor at 100 PPM hydrogen gas concentration.

To check the repeatability of the sensor, samples have been tested four times for 100 PPM H_2_ gas concentration at room temperature and the corresponding analysis has been shown in Fig. 12. The repeatability of the sensor has been tested inside the closed chamber equipped with PID controlled electrical heater. After every cycle, the heater is activated at 80 °C temperature. All the tested results are analogous which shows the repeatability as well as reproducibility in nature.

**Figure 13:**
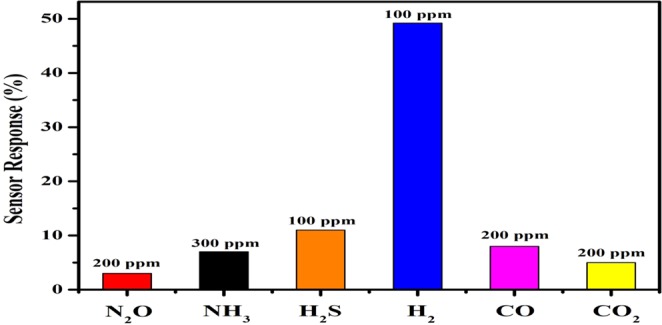
Selectivity of the 0.90(PVDF) − 0.10[0.75(SnO_2_) − 0.25 rGO] nanocomposite gas sensor towards various target gases.

The rGO/SnO_2_/PVDF nanocomposite sensor has been tested for different gases (Nitrous oxide (N_2_O), Ammonia (NH_3_), Hydrogen sulfide (H_2_S), Carbon monoxide (CO), and Carbon dioxide (CO_2_)) and for different concentrations at room temperature which are shown in Fig. 13. The obtained results proclaim that the proposed PVDF based nanocomposite sensor is very much selective for hydrogen gas.

**Figure 14:**
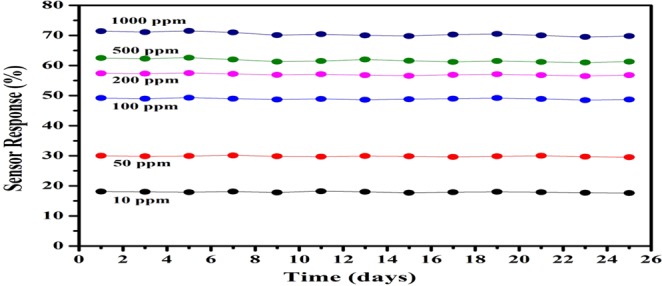
Time dependent stability of the 0.90(PVDF) − 0.10[0.75(SnO_2_) − 0.25 rGO] nanocomposite sensor for 10, 50, 100, 200, 500 and 1000 ppm H_2_ gas concentration.

Stability is also an important parameter which specifies the durability for all devices^34,35^. The proposed PVDF nanocomposite based gas sensor has been tested at room temperature for about a month on alternate days with different concentrations of hydrogen gas. The device shows little variation in sensor response and it has been shown in Fig. 14.

**Figure 15:**
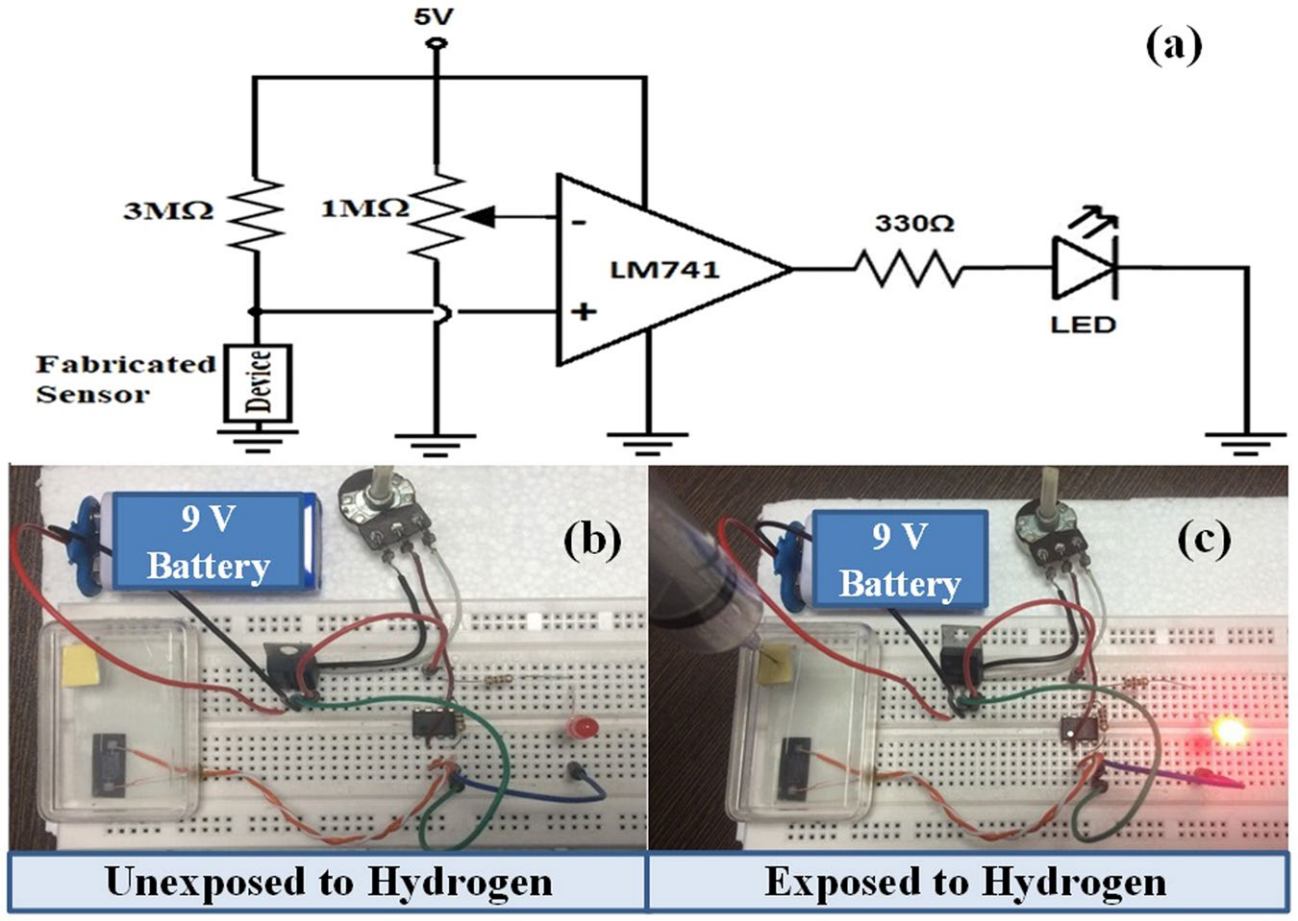
Real time demonstration of the proposed sensor, **(a)** circuit diagram, **(b**,**c)** are the sensor prototype in absence and presence of the hydrogen gas respectively.

The prototype of the proposed sensor with circuit diagram has been shown in Fig. 15. We have demonstrated the real time gas detection of the sensor by using an electronic circuit configuration. This assembly consists of comparator circuit by using LM 741 operational amplifier IC. The comparator is mainly designed to compare voltages at inputs. Therefore, we can decide the input terminal having larger voltage. In the comparator, one input is connected to potentiometer (1 MΩ) which serves as the reference voltage. The other input is connected to the proposed sensor via a fixed 3 MΩ resistor. In the absence of hydrogen gas, sensor module will show constant resistance and hence the LED will be in OFF condition. When the hydrogen gas will be exposed in the sensor assembly, then due to variation in resistance of the sensor, LED glows. The real time gas detection validates that the proposed model can be deployed in open environment for optimum sensor characteristics.

The nanocomposite of rGO, SnO_2_, and PVDF material have been reported first time, which is flexible, non-toxic, and environmentally stable. The proposed nanocomposite thick film of 0.90(PVDF) − 0.10[x(SnO_2_) − (1 − x)rGO] with different weight percentages (x = 0, 0.15, 0.30, 0.45, 0.6, 0.75, 0.90 and 1) have been prepared by hot press method, in which the x = 0.75 composition gave the best response when used as the H_2_ gas sensor. For 100 PPM hydrogen gas concentration, the sensor response for rGO/PVDF, SnO_2_/PVDF and rGO/SnO_2_/PVDF was 13.5, 23 and 49.2% respectively at room temperature. This indicates that the sensor response of rGO/SnO_2_/PVDF is about 2 times and 3.5 times more than SnO_2_/PVDF and rGO/PVDF nanocomposite respectively. The proposed nanocomposite also shows good repeatability and reproducibility. The nanocomposite also has been tested for various gases such as N_2_O, NH_3_, H_2_S, H_2_, CO, and CO_2_ and found that it is very much selective for hydrogen gas at room temperature. The rGO/SnO_2_/PVDF nanocomposite shows a detection limit up to 500 PPB H_2_ gas concentration and the testing result reveals that it has the best sensing ability compared to that of PVDF, rGO/PVDF, and SnO_2_/PVDF nanocomposite thick film at low gas concentration. The comparative analysis of different reported polymer based gas sensor towards the H_2_ gas has been represented in Table 1.

**Table 1:** Comparison of Sensing Parameters for various Polymer based Thick Film Towards H_2_ Gas.

Sample	Sensor Response (%)	Temperature (°C)	Gas Concentration (PPM)	Response/Recovery Time (sec)	Reference
rGO/SnO_2_/PVDF	49.2	RT	100	34 s/142 s	This work
rGO/SnO_2_/PVDF	71.4	RT	1,000	52 s/242 s	This work
PMMA/Pd NP/SLG	66	RT	20,000	108 s/331 s	^36^
rGO/Pd NP/PANI	25	RT	10,000	20 s/50 s	^37^
PAni/Au/PtO_2_	4	RT	4,000	−/−	^38^
PPy-modified Pd/Nafion	68	RT	3,500	300 s/−	^39^

## Conclusions

In this study, the thick films of nanocomposite of 0.90(PVDF) − 0.10[x(SnO_2_) − (1 − x)rGO] with different weight percentages (x = 0, 0.15, 0.30, 0.45, 0.6, 0.75, 0.90 and 1) have been fabricated by using hot press method and an interdigitated pattern of chromium have been deposited by using E-beam evaporation system. The crystal structure, microstructure and electrical properties of PVDF based nanocomposites have been discussed in detail. The response of the sensor has been tested for different H_2_ gas concentration at room temperature. Experimental results reveal that for the given atmospheric condition, 0.90(PVDF) − 0.10[0.75(SnO_2_) − 0.25rGO] nanocomposition gives better response compare to other compositions, especially at low H_2_ gas concentration. The sensor response of 49.2 and 71.4% with response time 34 sec and 52 sec for 100 PPM and 1000 PPM H_2_ gas concentration respectively have been obtained for 0.90(PVDF) − 0.10[0.75(SnO_2_) − 0.25 rGO] nanocomposite. This polymer based tertiary nanocomposite thick films will aid researchers to explore a new route to develop flexible, reliable and high-performance sensor for gas sensing applications.
